# STRESS PROCESSES IN AGING: CONSIDERING SOCIAL CONTEXT AND SELF-PERCEPTIONS

**DOI:** 10.1093/geroni/igae098.2100

**Published:** 2024-12-31

**Authors:** Kyrsten Hill

**Affiliations:** Chair

## Abstract

How individuals perceive, appraise, and cope with their stressors holds dramatic influence on whether they experience positive aging trajectories. However, stress-related processes are inherently contextual, reflecting an interaction between the person and the environment. The current series of talks considers how individuals deal with stress across different time scales. The first two talks will consider stress in a social context, evaluating the roles of daily loneliness and interpersonal stressors. First, Kang will describe findings from an EMA study suggesting a reciprocal relationship between momentary loneliness and cognitive performance. Second, Ong will report on an EMA study examining the relationship between self-perceptions of aging and daily social interactions. The final two talks will consider adults’ perceptions of their ability to deal with stressors, considering control beliefs and coping processes over the short- and long-term. Third, Neupert will discuss how daily stressors and daily control beliefs are associated with the perceived rate at which people feel that they are aging. Finally, Kurth will discuss how coping strategies change longitudinally over 10 years and how the rate and direction of change depends on age. Together, this symposium highlights how stress processes influence aging across individuals, time courses, and contexts.

